# Development of 4H-pyridopyrimidines: a class of selective bacterial protein synthesis inhibitors

**DOI:** 10.1186/2191-2858-2-5

**Published:** 2012-02-16

**Authors:** Joseph W Guiles, Andras Toro, Urs A Ochsner, James M Bullard

**Affiliations:** 1 Replidyne, Inc., Louisville, CO, USA; 2 Chemistry Department, SCIE. 3.320, The University of Texas-Pan American, 1201 W. University Drive, Edinburg, TX 78541, USA; 3 CedarburgHauser Pharmaceuticals, Denver, CO, USA; 4 Mannkind Corporation, Valencia, CA, USA; 5 Crestone, Inc., Boulder, CO, USA

**Keywords:** antibiotic, drug discovery, structure-activity relationship (SAR), protein synthesis, inhibitor, *Staphylococcus aureus*, *Streptococcus pneumoniae*

## Abstract

**Background:**

We have identified a series of compounds that inhibit protein synthesis in bacteria. Initial IC_50_'s in aminoacylation/translation (A/T) assays ranged from 3 to14 μM. This series of compounds are variations on a 5,6,7,8-tetrahydropyrido[4,3-*d*]pyrimidin-4-ol scaffold (e.g., 4H-pyridopyrimidine).

**Methods:**

Greater than 80 analogs were prepared to investigate the structure-activity relationship (SAR). Structural modifications included changes in the central ring and substituent modifications in its periphery focusing on the 2- and 6-positions. An A/T system was used to determine IC_50 _values for activity of the analogs in biochemical assays. Minimum inhibitory concentrations (MIC) were determined for each analog against cultures of *Enterococcus faecalis*, *Moraxella catarrhalis, Haemophilus influenzae, Streptococcus pneumoniae*, *Staphylococcus aureus*, *Escherichia coli tolC *mutants and *E. coli *modified with PMBN.

**Results:**

Modifications to the 2-(pyridin-2-yl) ring resulted in complete inactivation of the compounds. However, certain modifications at the 6-position resulted in increased antimicrobial potency. The optimized compounds inhibited the growth of *E. faecalis, M. catarrhalis, H. influenzae*, *S. pneumoniae*, *S. aureus*, *E. coli tolC*, mutants and *E. coli *modified with PMBN with MIC values of 4, ≤ 0.12, 1, 2, 4, 1, 1 μg/ml, respectively. IC_50 _values in biochemical assay were reduced to mid-nanomolar range.

**Conclusion:**

4H-pyridopyrimidine analogs demonstrate broad-spectrum inhibition of bacterial growth and modification of the compounds establishes SAR.

## 1. Background

Bacterial infections continue to represent a major worldwide health hazard. Our health care systems are increasingly confronted with drug-resistant hospital and community-acquired infections [1]. With the recent emergence of numerous, clinically important, drug-resistant bacteria including *Staphylococcus aureus*, *Streptococcus pneumoniae*, *Enterococcus faecalis*, *Mycobacterium tuberculosis*, enhanced-spectrum β-lactamase producing *Escherichia coli *and *Klebsiella sp*. and *Pseudomonas aeruginosa*, an emergency is becoming apparent. Antibacterials kill bacteria by interfering with processes of cellular function that are essential for their survival. The majority of clinically important antibiotics target the ribosome and protein synthesis in general [2,3] and most of these are naturally occurring antibiotics or derivatives of naturally occurring antibiotics [4,5].

**Figure 1:**
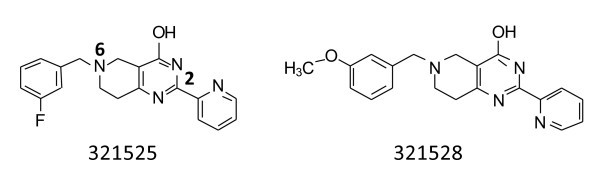
**The two most potent compounds coming out of the original A/T HTS**.

We have developed an aminoacylation/translation (A/T) system for screening for inhibitors of protein synthesis and in high throughput screens (HTS) of focused chemical compounds we identified a class of selective bacterial protein synthesis inhibitors, 5,6,7,8-tetrahydropyrido[4,3-*d*]pyrimidin-4-ol (e.g., 4H-pyridopyrimidine) [6]. Two compounds, 321525 and 321528 (Figure 1), were found to exhibit the greatest inhibitory activity in the initial HTS using the A/T assays and subsequently antibacterial activity was confirmed against *S. pneumoniae*, *S. aureus*, and *E. coli tol*C mutants. The compounds 321525 and 321528 were retested in the A/T assay and inhibited protein synthesis with IC_50_'s of 2.8 and 1.2 μM, respectively. Minimum inhibitory concentrations (MIC) were determined for a panel of bacteria including *E. faecalis, Moraxella. catarrhalis, Haemophilus influenzae, S. pneumoniae*, *S. aureus*, *E. coli tolC *mutants, and *E. coli *modified with PMBN. The MIC of 321525 and 321528 against these pathogens was 32, 0.25, 4, 8, 32, 8, 32, and 128, 2, 8, 32, > 128, 128, 32 μg/ml, respectively [6]. The inhibitory activity of these two compounds encouraged us to initiate structure-activity relationship (SAR) studies. Previously, minimum bactericidal concentration testing of the 4H-pyridopyrimidines initially indicated that the compounds were bactericidal against *H. influenzae*, but only bacteriostatic against *S. pneumoniae *[6,7]. Also, we previously conducted macromolecular synthesis (MMS) assays to test compounds to determine if RNA, DNA, or protein synthesis was inhibited in bacterial cultures. Assays were carried out in cultures containing the *E. coli tol*C mutant and also in cultures of *S. aureus*. The MMS data for two representative compounds, REP323219 and REP323370, indicate that the 4H-pyridopyrimidines are specific inhibitors of protein synthesis in the cell [6]. We report here the results of an in-depth SAR study of the inhibitory compound series.

## 2. Methods and materials

The original hit compounds were from a chemical compound library containing 2100 compounds from Asinex (Moscow, Russia). All analogs of the original hit compounds were prepared by Asinex. Biochemical analysis and determination of IC_50 _values of the original compounds and testing of the analogs were carried out using the A/T assay as described [6]. Broth microdilution MIC testing was performed in 96-well microtiter plates according to Clinical Laboratory Standards Institute (CLSI; formerly NCCLS) document M7-A6 [8]. MIC values were determined for *E. faecalis, M. catarrhalis, H. influenzae, S. pneumoniae*, *S. aureus*, *E. coli tolC *mutants, and *E. coli *modified with PMBN. MMS assays were performed in cultures of *E. coli tol*C mutants as described [6,9].

## 3. Results and discussion

**Figure 2:**
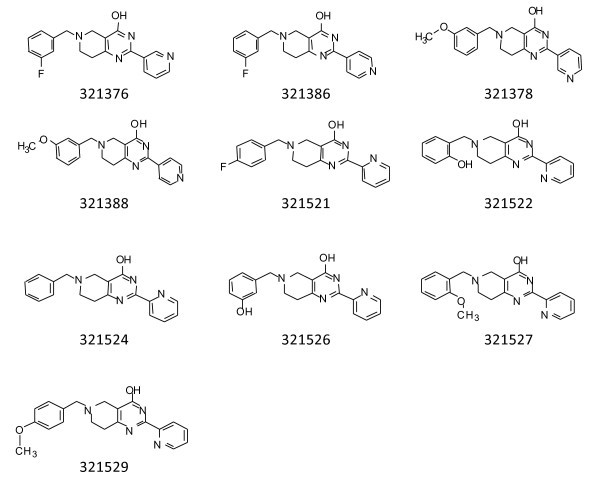
**Additional compounds identified in the original compound library**.

We re-evaluated 321376, 321386, 321388, 321378, 321521, 321522, 321524, 321526, 321527, and 321529 from the initial library obtained from Asinex (Figure 2). These compounds are similar to 321525 and 321528. The first four were identical to the original compounds with the exception that the nitrogen in the 2-pyridin-2-yl was walked around the pyridine ring to the 3-yl and 4-yl positions (Figure 2). Movement of the nitrogen resulted in complete loss of inhibitory activity, both biochemical and biological (Table 1). Similarly, in the final six compounds, the effect of the 3-fluoro- and 3-methoxy-benzyl substitution at the 6-position were tested by removing or walking around these functional groups on their parent 6-benzyl group. These initial compounds also contained 2- and 3-hydroxy substitutions at the 6-position. In the initial HTS, these compounds exhibited activity, but fell below the cutoff that defined a hit compound. When these compounds were re-assayed in triplicate they exhibited similar IC_50 _values as the original two compounds (Table 1). When tested against the panel of bacteria, these compounds also showed similar bacterial growth inhibition as 321525 and 321528, with the exception of 321526 which exhibited little or no anti-bacterial activity.

**Table 1:** IC_50 _and MIC values of compounds against pathogenic bacteria

Compound	IC_50 _(μM)	E. fae* (μg/ml)	*M. cat *(μg/ml)	*H. flu *(μg/ml)	*S. pneumo *(μg/ml)	*S. aureus *(μg/ml)	*E. coli tolC *(μg/ml)	*E. coli +*PMBP (μg/ml)
321376	> 300	N/D	N/D	N/D	N/D	> 128	> 128	N/D
321578	> 300	> 128	> 128	> 128	> 128	> 128	> 128	> 128
321386	> 300	> 128	> 128	> 128	> 128	> 128	> 128	> 128
321388	> 300	> 128	> 128	> 128	> 128	> 128	> 128	> 128
321521	2.4	16	0.5	4	16	128	16	64
321522	2.6	16	1	2	16	128	32	64
321524	2.0	16	1	4	16	128	16	64
321526	3.5	> 128	64	> 128	> 128	> 128	> 128	> 128
321527	1.8	64	2	16	32	> 128	64	> 128
321529	2.6	128	2	8	32	> 128	32	128
323191	> 300	128	4	32	32	> 128	128	64
323192	> 300	128	4	64	32	> 128	128	64
323193	4.1	> 128	> 128	> 128	> 128	> 128	> 128	> 128
323194	3.4	64	1	8	8	64	16	16
323195	1.5	64	2	16	32	64	32	16
323196	1.6	32	128	> 128	> 128	> 128	> 128	> 128
323197	4.4	> 128	16	32	128	> 128	> 128	> 128
323198	2.7	> 128	1	8	16	> 128	32	64
323199	1.1	128	1	16	16	128	32	32
323200	0.4	> 128	> 128	> 128	> 128	> 128	> 128	> 128
323201	2.1	> 128	> 128	> 128	> 128	> 128	> 128	> 128
323202	4.3	32	0.25	4	4	16	8	32
323203	2.0	64	1	8	4	16	8	16
323204	2.1	> 128	64	32	128	> 128	> 128	> 128
323205	> 300	128	4	16	32	> 128	64	64
323206	> 300	> 128	> 128	> 128	> 128	> 128	> 128	> 128
323207	> 300	> 128	> 128	> 128	> 128	> 128	> 128	> 128
323208	2.7	> 128	4	64	32	> 128	64	64
323209	1.5	> 128	4	64	32	> 128	64	64
323210	3.2	> 128	> 128	> 128	> 128	> 128	> 128	> 128
323211	1.6	> 128	64	> 128	> 128	> 128	> 128	> 128
323212	3.4	128	1	> 128	4	64	8	16
323213	2.4	> 128	> 128	> 128	> 128	> 128	> 128	> 128
323214	> 300	> 128	> 128	> 128	> 128	> 128	> 128	> 128
323215	2.1	> 128	128	64	> 128	> 128	> 128	> 128
323216	1.1	32	≤0.12	2	4	16	8	8
323217	4.8	> 128	32	32	> 128	> 128	> 128	> 128
323218	0.24	> 128	4	16	32	> 128	128	64
323219	3.0	32	≤0.12	4	4	16	8	16
323220	2.1	32	≤0.12	2	4	16	8	8
323221	> 300	> 128	> 128	> 128	> 128	> 128	> 128	> 128
323222	> 300	> 128	> 128	> 128	> 128	> 128	> 128	> 128
323223	14.7	> 128	4	32	32	> 128	128	64
323224	4.9	> 128	16	8	> 128	> 128	> 128	128
323225	17.1	> 128	2	8	128	> 128	> 128	> 128
323226	8.0	> 128	64	> 128	> 128	> 128	> 128	> 128
323227	0.37	> 128	8	32	64	> 128	64	> 128
323228	0.30	64	0.5	4	8	31	16	16
323229	0.53	64	0.5	8	8	64	16	16
323230	2.0	32	0.5	8	8	32	16	16
323231	5.9	128	128	128	128	> 128	> 128	> 128
323232	3.2	> 128	8	> 128	> 128	> 128	128	> 128
323233	> 300	128	4	16	32	> 128	64	64
323234	5.8	128	16	128	128	> 128	> 128	64
323235	37.9	> 128	8	64	64	> 128	128	128
323236	7.8	> 128	> 128	> 128	> 128	> 128	> 128	> 128
323237	2.8	64	0.5	4	8	32	8	16
323238	2.1	> 128	16	32	> 128	> 128	> 128	> 128
323239	3.0	64	8	64	> 128	> 128	64	128
323338	64.4	16	≤0.12	2	4	16	4	8
323339	19.0	64	≤0.12	8	4	32	2	2
323340	> 300	16	≤0.12	1	2	4	1	2
323341	41.4	32	≤0.12	2	4	16	8	16
323342	28.9	32	≤0.12	2	2	32	4	16
323343	8.24	32	≤0.12	1	2	8	4	2
323344	11.6	32	≤0.12	4	2	8	1	4
323345	> 300	> 128	32	64	128	> 128	128	128
323353	1.7	> 128	8	32	128	> 128	128	128
323354	21.6	> 128	16	> 128	> 128	> 128	> 128	> 128
323355	32.1	> 128	4	64	64	128	64	64
323356	> 300	128	8	64	128	> 128	128	> 128
323365	2.27	16	≤0.12	4	4	8	4	4
323366	2.47	8	≤0.12	4	2	8	4	2
323367	1.89	4	≤0.12	1	2	8	2	8
323368	0.47	> 128	> 128	> 128	> 128	> 128	> 128	> 128
323369	2.01	64	4	16	64	128	64	32
323370	0.28	16	≤0.12	2	4	16	8	8
332052	> 300	> 128	32	> 64	> 128	> 128	> 128	> 128
332053	> 300	> 128	> 128	> 64	> 128	> 128	> 128	> 128
332054	7.4	8	≤0.12	1	2	4	2	1
332055	16.4	64	32	16	128	> 128	128	64
332057	101	> 128	> 128	> 128	> 128	> 128	> 128	> 128
332058	0.56	16	≤0.12	0.5	2	16	4	4

*E. faecalis, M. catarrhalis, H. influenzae, S. pneumoniae, S. aureus, *E. coli tolC *mutants, and *E. coli *modified with PMBN

**Figure 3:**
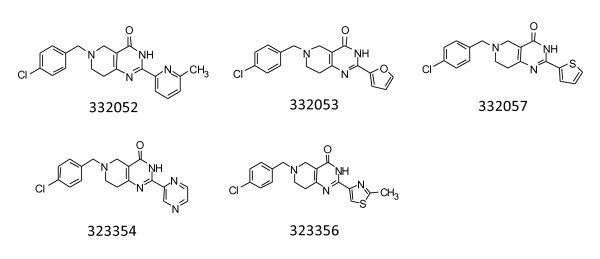
**Compounds in which the pyridine ring attached to the central scaffold/core in the 2-position was modified**.

To determine how critical the structure of the 2-pyridin-2-yl ring was to the activity of the compound series, we introduced two moderate changes to 321521 (Figure 3). First, a methyl group was added next to the nitrogen in the pyridine ring (332052) and second, the pyridine ring was replaced with a pyrazine ring (323354) (Figure 3). The compound 323354 reduced potency tenfold and 332052 completely abolished activity in the A/T assay. Both compounds lost anti-bacterial activity against all bacteria in the panel, except against *M. catarrhalis *where the MIC values increased 120- and 60-fold, respectively. Next, the pyridine ring at the 2-position was replaced with a furan (332053), or a thiophene (332057), or a methyl thiazole (323356) (Figure 3). The furan and methyl thiazole replacements abolished all biochemical activity in the A/T assay, while biochemical activity of the thiophene replacement was reduced over 40-fold. Only the methyl thiazole replacement exhibited slight antibacterial activity. More analogs will be required to complete a comprehensive study of this part of the compound series, but from these preliminary results all changes were observed to be deleterious to the activity of the compound series.

**Figure 4:**
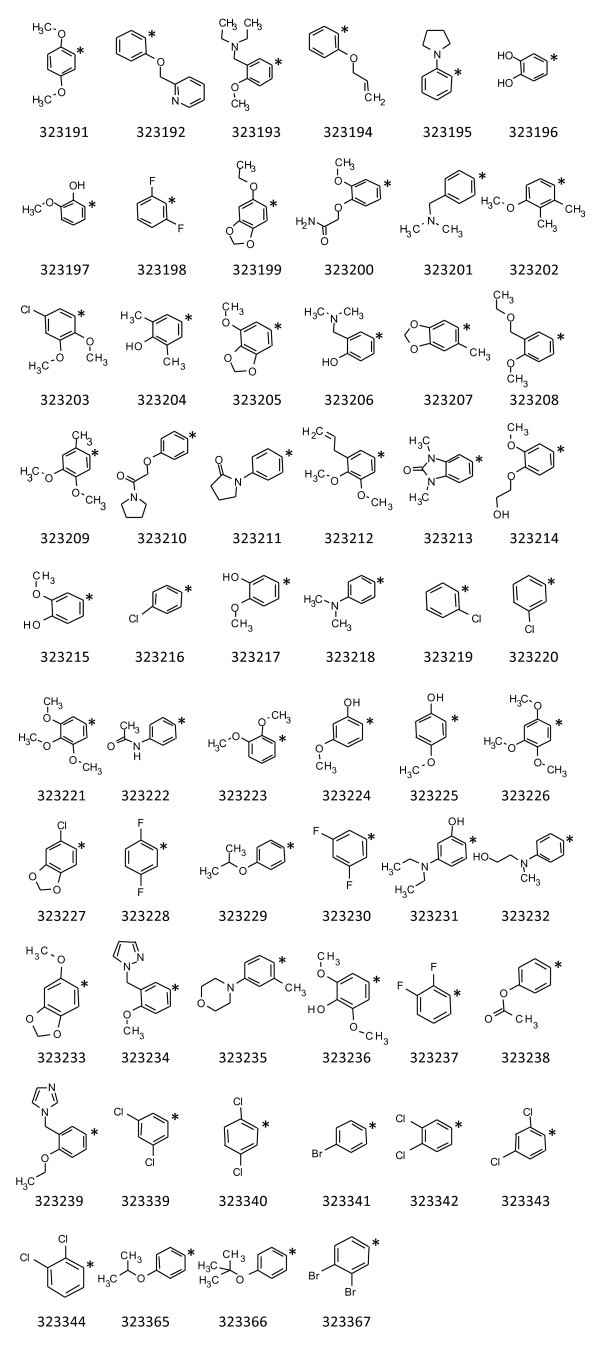
**Compounds in which the benzene ring attached at the 6-position of the core structure was modified**. Asterisk represents the point of attachment to the core structure.

Fifty-eight new compounds were prepared in which the phenyl ring at the 6-position of the core structure was modified (Figure 4). In the instances that the modification contained a hydroxyl or a carbonyl group (323196, 323197, 323200, 323204, 323206, 323210, 323211, 323213, 323214, 323215, 323217, 323222, 323224, 323225, 323231, 323232, 323236, 323238), the biochemical potency was maintained or increased; however, in each case the microbiological inhibition was lost (Table 1). For example, compound 323200 in which the benzene ring was modified to a 2-(2-methoxyphenoxy) acetamide, the IC_50 _was improved three to sevenfold yet the MIC was above maximum testing concentrations for the entire panel of bacteria. When the phenyl ring contained multiple methyl or methoxy substitutions (323191, 323202, 323209, 323221, 323223, 323226) an overall increase in the IC_50 _and MIC values (decrease in potency) was observed. In the case of 323221, the 3,4,5-trimethoxy substitution abolished both biochemical and microbiological activities.

Further five-member ring (323195, 323234, 323239) or six-member ring (323192, 323235) substitutions at the 6-position decreased the potency with the exception of compound 323195 in which the benzene ring was replaced with 1-phenylpyrrolidine. In this case, the activity was observed to be comparable to the original two compounds. Next, five member-ring structures were fused to the benzene ring to form five benzodioxoles (323199, 323205, 323207, 323227, 323233) and one benzimidazol (323213) (Figure 4). These compounds all tended to decrease potency of the compound series with the exception of 323227. When the benzene ring was replaced with 5-chloro-1,3-benzodioxole in 323227 a three to sevenfold improvement in the IC_50 _was observed.

A 4-dimethylamino substitution (323218) to the phenyl ring resulted in a five and tenfold improvement in the IC_50 _but an increase was observed in the MIC for all bacteria tested. When the 4-dimethylamino of 323218 was modified to a 4-dimethylaminomethyl group (323201) or to a 3-diethylaminomethyl group (323193), biochemical activity was maintained however all antibacterial activity was lost.

The most moderate modification to the benzene ring at the 6-position was the attachment of one or two halogens to the ring structure. These changes resulted in increased potency of the compound series in inhibition of bacterial growth. However, not all of this set of compounds improved the IC_50 _values. When two fluorine atoms (323198, 323228, 323230, and 323237) or one chlorine atom (323216, 323219, and 323220) were walked around the phenyl ring at the 6-position on the core structure, the biochemical potency was maintained or increased. However, the biochemical potency was decreased with compounds in which two chlorine atoms (323339, 323340, 323342, 323343, and 323344) were walked around the phenyl ring. Compounds containing a 3-bromobenzene or a 2,3-dibromobenzene at the 6-position (323341, 323367) also decreased biochemical potency. Even though a moderate decrease in biochemical potency was observed for a subset of these compounds as a whole they exhibited some of the greatest increases in potency for inhibiting bacterial growth. The 2,3-dimethoxy-5-chloro substitution in 323203 maintained biochemical and enhanced antibacterial potency.

The remaining four compounds (323194, 323208, 323212, and 323229) contained 2-(ethenyloxy)benzene, 2-(ethoxymethyl)-3-(methoxybenzene, 2,3-dimethoxy-4(prop-2-en-1-yl)benzene and 3-(propan-2-yl ether)benzene at the 6-position, respectively. Variations of alkyl, alkenyl, alkoxyl, and alkenoxy groups exhibited no extreme shifts in either the IC_50 _or MIC values, although all four were potent compounds.

**Figure 5:**
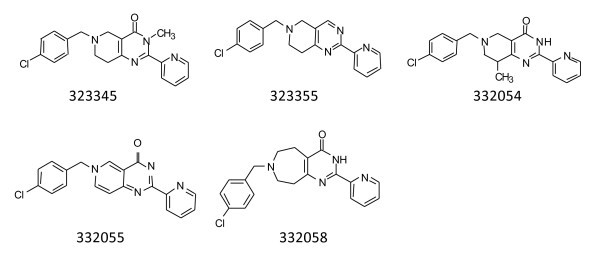
**Compounds in which the central scaffolds/cores were modified**.

Next, based on compound 323216 (Figure 4), changes to the 5,6,7,8-tetrahydropyrido[4,3-*d*]pyrimidin-4-ol core were implemented (Figure 5). Removal of the carbonyl group from the pyrimidine ring of the core (323355) resulted in moderate increase of both IC_50 _and the MIC values. Attachment of a 3-methyl group onto the tetrahydropyrido[4,3-*d*]pyrimidin (323345) abolished most biochemical and antibacterial activities, whereas addition of an 8-methyl group (332054) enhanced antibacterial activity. Oxidation of the tetrahydropyrido[4,3-d]pyrimidin core (332055) resulted in a negative effect on the activity of the compounds series. Ring enlargement to tetrahydro-3H-pyrimido[5,4-c]azepin (332058) had profound effects on both IC_50 _and MIC values. The IC_50 _was improved to 0.56 μM and the MIC values determined for different bacteria in the panel were improved to the lowest levels observed for any compound tested.

**Figure 6:**
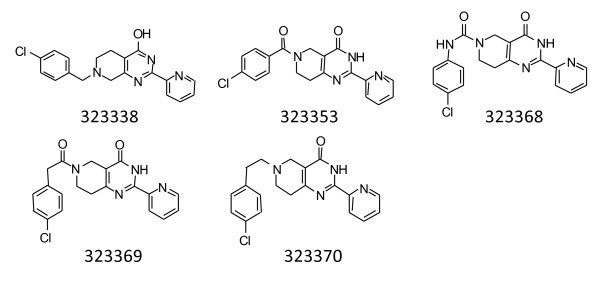
**Compounds in which the linker connecting the benzene ring attached at the 6-position of the core structures were modified**.

Finally, changes were made in the linker tethering the phenyl ring to the 6-position of the core scaffold (Figure 6). First, the point of attachment of the linker was changed from the 6-position to the 5-position (323338). This resulted in a significant increase in the IC_50 _but a slight improvement in the MIC values. Next, the methylene linker was replaced with a carbonyl carbon (323353) or a carboxamido linker (323368). The insertion of the carbonyl carbon resulted in decreased potency in bacterial cultures. The amide group resulted in an improved IC_50_, but a complete loss of antibacterial activity was observed. Next, the length of the carbon linker was increased by the addition of a carbonyl carbon (323369) or with an additional carbon (323370). The addition of the carbonyl carbon significantly increased the MIC. Insertion of an additional carbon however improved the IC_50 _to one of the lowest values recorded and maintained low MIC values.

## 4. Conclusion

In summary, this library of compounds allows a determination of what kind and where changes in the compound series will be allowed. It is obvious that almost any change in the pyridine ring at the 2-position of the core will not be tolerated. Changes to the benzene ring tethered to the 6-position of the core are permitted but tend to increase potency when they are simple in nature. It is possible to lengthen the 6-linker, but adding functional groups to the linker may impede antibacterial activity. Of the compounds tested, the IC_50 _was improved to sub-micromolar values with seven compounds (Table 2). Most of these compounds maintained or increased MIC's when compared to 321525 and 321528; however, one compound completely abolished antibacterial activity (Table 2). In some instances, there was a complete disconnect between IC_50 _and MIC values. Compound 323340 displayed a good antibacterial activity; however, it had no inhibitory effect in the A/T assay suggesting a different mechanism of inhibition (Table 3). When comparing biochemical inhibitory activity and antibacterial activity of the compounds, only two compounds (323370 and 332058) appear in both tables. It is possible that compounds with good biochemical activity that lack antibacterial potency may not be able to enter the bacterial cells, or are effluxed or modified to an inactive state by the bacteria. Also, compounds with good MIC but poor IC_50 _values may exhibit a secondary mode of action that leads to the observed antibacterial activity.

**Table 2:** Comparison of good IC_50 _to MIC values

Compound	IC_50 _μM	MIC
323218	0.24	+
323227	0.37	+
323228	0.30	++
323229	0.53	++
323368	0.47	-
323370	0.28	+++
332058	0.56	+++

+, poor; ++, moderate; +++, good antibacterial activity.

**Table 3:** Comparison of good MIC to IC_50 _values

Compound	MIC	IC_50 _μM
323338	+++	64.4
323339	+++	19.0
323340	+++	> 300
323341	+++	41.4
323342	+++	28.9
323343	+++	8.24
323344	+++	11.6
323365	+++	2.27
323366	+++	2.47
323367	+++	1.89
323370	+++	0.28
332054	+++	7.40
332058	+++	0.56

**Figure 7:**
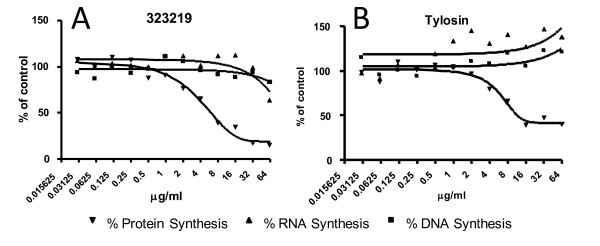
**An MMS assay showing inhibition of DNA, RNA, and protein synthesis in *E. coli tol*C cultures**. MMS assays in which (A) 323219 and (B) tylosin were titrated into the assays. In these assays, the IC_50 _for 323219 and tylosin were calculated to be 6.2 and 11.6 μg/ml, respectively. Black triangle represents the percent of RNA synthesis, black square represents the percent of DNA synthesis, and black inverted triangle represents the percent of protein synthesis inhibited.

We have demonstrated specific inhibition of protein synthesis in *E. coli tolC *cells for a number of the compounds described above, including 323202, 323203, 323216, 323219, and 323220. These results indicate that the types of modifications in these compounds are tolerated with regard to maintaining the specific mode of action. When compared with a known inhibitor (tylosin) of bacterial protein synthesis, 323219 exhibited similar inhibition profiles in MMS assays (Figure 7). The other compounds tested yielded similar results.

## Competing interests

The authors declare that they have no competing interests.
